# Whole-body vibration training on functional capacity, vascular function, and glycemic control in individuals with type 2 diabetes and peripheral arterial disease: protocol for a randomized controlled trial

**DOI:** 10.3389/fendo.2026.1734560

**Published:** 2026-02-26

**Authors:** Sothida Nantakool, Busaba Chuatrakoon, José G. B. Derraik, Silmara Gusso

**Affiliations:** 1 Integrated Neuro-Musculoskeletal, Chronic Disease, and Aging Research Engagement Center (ICARE Center), Department of Physical Therapy, Faculty of Associated Medical Sciences, Chiang Mai University, Chiang Mai, Thailand; 2 Environmental-Occupational Health Sciences and Non-Communicable Diseases Research Center, Research Institute for Health Sciences, Chiang Mai University, Chiang Mai, Thailand; 3 Department of Women’s and Children’s Health, Uppsala University, Uppsala, Sweden; 4 Department of Paediatrics: Child and Youth Health, Faculty of Medical and Health Sciences, University of Auckland, Auckland, New Zealand; 5 Department of Exercise Sciences, Faculty of Science, University of Auckland, Auckland, New Zealand

**Keywords:** blood flow, functional capacity (FC), peripheral arterial disease (PAD), RCT, type 2 diabetes, vibration

## Abstract

**Background:**

Type 2 diabetes and peripheral arterial disease contribute to long-term disability. Walking-based exercise improves related symptoms, but adherence is limited by low fitness and leg pain. Whole-body vibration stimulates muscle contractions and increases limb and microvascular blood flow, which can mimic the impact of walking. Prior studies suggest improved endothelial function, reduced arterial stiffness, and lower glycated hemoglobin following vibration therapy; however, its effects on functional capacity in this group remain unclear.

**Aims:**

To investigate whether whole-body vibration impacts functional capacity achieved with supervised treadmill walking, as measured by incremental shuttle walk test distance with a pre-specified non-inferiority margin.

**Study design:**

Randomized controlled trial of 48 participants (24 per group) aged 50 years or older with ankle-brachial index 0.9 or less or toe-brachial index 0.7 or less and Fontaine stage I to IIb will be included. Participants will receive whole-body vibration or supervised treadmill walking for 12 weeks, three sessions per week. Whole-body vibration will be delivered using a Galileo plate. Analyses will follow the intention-to-treat principle using a general linear model adjusted for age, sex, peripheral arterial disease category, diabetes duration, and baseline incremental shuttle walk test distance.

**Primary outcome:**

Distance in the incremental shuttle walk test.

**Secondary outcomes:**

Ankle-brachial index, toe-brachial index, brachial artery flow mediated dilation, glycated hemoglobin, SF-36 health related-quality of life, and Peripheral Artery Questionnaire scores. Adverse events will be monitored.

**Implications for clinical practice:**

If efficacy is demonstrated, this intervention could serve as a clinic-based exercise option to improve mobility, vascular function, and glycemic control in this population.

**Trial registration:**

Thai Clinical Trials Registry - TCTR20251020003.

## Introduction

Diabetes, particularly type 2 diabetes (T2D), is a major non-communicable disease that poses considerable public health challenges globally, including in Thailand. It is well-documented that approximately 90% of individuals with type 2 diabetes develop macrovascular complications, most notably cardiovascular diseases such as coronary artery disease, stroke, and peripheral arterial disease (PAD), which collectively contribute to increased morbidity, disability, and mortality (1).

PAD is a common and clinically significant vascular disorder in diabetic populations, primarily affecting the lower extremities (2). The condition is mainly driven by atherosclerosis, a process accelerated by chronic hyperglycemia, which induces arterial injury through various mechanisms, including platelet aggregation, chronic inflammation, endothelial dysfunction, and vascular smooth muscle cell dysfunction (3). These hyperglycemia-mediated vascular changes promote progressive atherosclerotic narrowing of peripheral arteries, forming the pathological basis of PAD (3). Hemodynamically, PAD is defined by diminished perfusion pressure. The exercise-related impact of PAD becomes especially pronounced in the lower-extremity arteries, where atherosclerotic stenosis limits the capacity to increase blood flow during physical activity, leading to an oxygen supply-demand mismatch and reversible ischemia (4). This reversible ischemia manifests clinically as intermittent claudication, a hallmark symptom of PAD in diabetes, characterized by exertional leg pain (4). Persistent ischemic burden contributes to functional impairment and may eventually lead to limb amputation, resulting in disability and mobility impairment, and reduced quality of life (5–8). Therefore, therapeutic strategies targeting improvement in arterial function are likely to enhance functional capacity.

Walking-based exercise training is a non-pharmacological treatment approach recommended for patients with PAD (9, 10). A meta-analysis showed that exercise can improve walking ability and enhance quality of life in PAD patients (11). However, despite the beneficial clinical effects of exercise training, poor exercise adherence seems to be common in patients with PAD (11, 12). Two key factors contributing to poor adherence are poor cardiorespiratory fitness (11) and low motivation due to leg symptoms (12). Thus, alternative exercise modalities that can overcome these limitations are needed.

Whole-body vibration therapy (WBV) is a relatively new therapeutic modality. It involves the use of a mechanically oscillating platform to deliver low- to high-frequency vibrations throughout the body inducing involuntary muscle contractions (13), stimulating limb and microvascular blood flow. A meta-analysis showed increased peripheral blood flow following acute bouts of WBV in healthy subjects (14). However, to our knowledge, no studies have investigated the therapeutic effects of WBV on limb and microvascular blood flow and glycemic control in individuals with both T2D and PAD. WBV-induced muscle contractions can enhance venous return and shear stress, which are recognized systemic hemodynamic stimuli. These physiological responses underpin potential systemic and local vascular effects (15, 16). Evidence suggests that WBV can reduce arterial stiffness in young women with obesity (17) and improve endothelial function response in elderly with cardiovascular disease (18). Furthermore, a reduction in hemoglobin A1C, a marker for long-term glycemic control, has been reported following WBV training in individuals with T2D (19).

Importantly, improvements in vascular function and glycemic control are not merely surrogate markers but have the potential to translate into enhanced functional capacity. Functional capacity is a clinically meaningful endpoint in individuals with PAD, as it reflects mobility, independence, and quality of life in this population. Despite these potential links, it remains unclear whether WBV improves functional capacity as a downstream effect of enhanced vascular function and potentially improved glycemic control in individuals with T2D and PAD. Therefore, the objectives of this study are (i) to examine the effects of WBV on functional capacity in individuals with T2D and PAD, (ii) to determine the effects of WBV on vascular function, glycemic control, and quality of life in this population.

## Methods and analysis

### Participants

The study will recruit participants from the Outpatient Clinic (Department 21) at the Maharaj Nakorn Chiang Mai Hospital (Chiang Mai, Thailand). Eligible patients who meet the following inclusion criteria will be invited to participate in the study:

1. Having a formal T2D diagnosis;
2. Having a formal PAD diagnosis (20):
  - ankle-brachial index (ABI) ≤0.9; OR
  - toe-brachial index (TBI) ≤0.7;
3. Having PAD severity I–IIb according to the Fontaine classification system, i.e., ranging from asymptomatic to severe claudication while walking (21);
4. Be aged ≥50 years.

Potential participants will be excluded from the study if meeting any of the following criteria:

1. Symptomatic chronic heart failure according to a New York Heart Association functional class of III-IV (22);
2. Recent history of cardiovascular events within the previous 6 months (e.g., myocardial infarction, stroke, thrombosis);
3. Having undergone vascular surgery within the previous 6 months;
4. Having any other medical conditions for which WBV would not be advisable, such as benign paroxysmal positional vertigo (BPPV);
5. Currently undergoing hemodialysis;
6. Ongoing pregnancy;
7. Inability to walk independently with both feet on the floor even with the use of assistance devices (e.g., crutches, walker, or cane);
8. Lower-limb amputees;
9. Having any cognitive impairment that would preclude the patient from following the WBV protocol appropriately and for the duration of the study, or from complying with the primary outcome assessment.

### Study design

**Figure 1:**
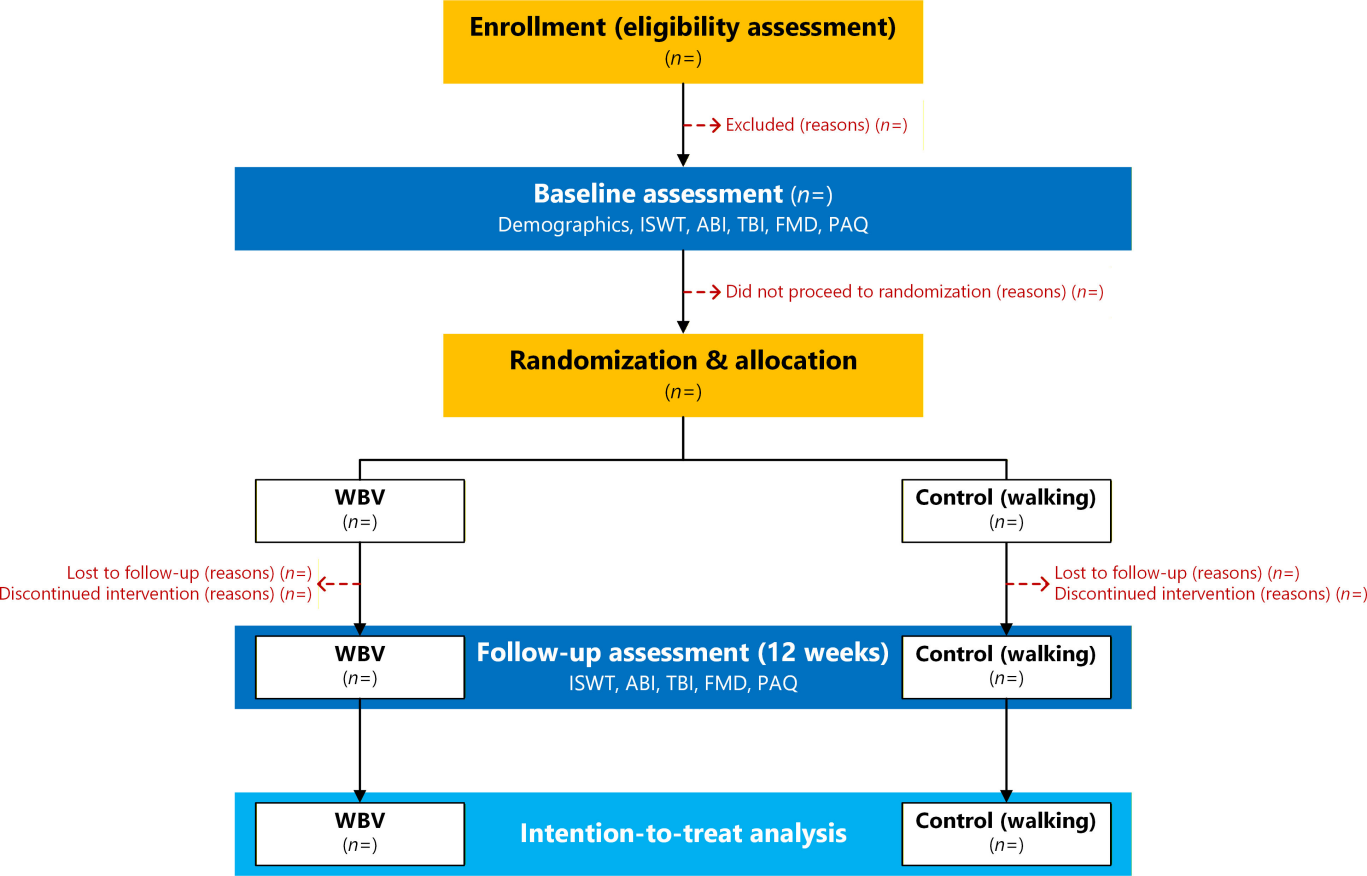
CONSORT flow diagram of participant progression through the trial. Flow of participants through enrollment (eligibility assessment and exclusions), baseline assessment, randomization, allocation to whole-body vibration training (WBV) or control (walking therapy), follow-up assessment (after 12 weeks), and intention-to-treat analysis. Assessments include demographics; functional capacity (Incremental Shuttle Walk Test, ISWT); vascular indices (ankle-brachial index, ABI; and toe-brachial index, TBI); endothelial function (flow-mediated dilation, FMD); and health-related quality of life (Peripheral Artery Questionnaire, PAQ). *n* indicates the number of participants at each stage.

This will be a randomized controlled trial, with participants randomized in a 1:1 ratio to either walking (control arm) or whole-body vibration (WBV; treatment arm) (**Figure 1**) using a computer-generated randomization sequence with variable block sizes of 2 and 4 (23). Randomization will be stratified by biological sex (male, female), ensuring that within each sex stratum the group allocation is balanced.

The intervention will consist of 12 weeks of clinic-based supervised WBV on a Galileo Basic vibration plate (Novotec Medical, Pforzheim, Germany). All participants will be able to access subsidized transport to and from the clinic for the duration of the treatment period. The intervention protocol will be adapted from previously developed and implemented WBV protocols (24), with adjustments informed by previous studies investigating glycemic control and leg blood flow in individuals with T2D (19, 25). Participants will perform WBV three times per week for 12 weeks at a frequency ranging from 15 to 27 Hz and an amplitude of 2 to 4 mm (19, 25) (**Table 1**). Each training session will last approximately 22–28 minutes and will consist of 5-minute warm-up involving stretching exercises, three WBV bouts lasting 3–5 minutes each separated by 1-minute rest periods (resulting in a total WBV exposure of 12–18 minutes), and a 5-minute cool-down with stretching exercises. The vibration frequency, amplitude, and bout duration will be progressively adjusted throughout the intervention in accordance with the progression scheme described in **Table 1**. Participants will need to stand on the plate while barefoot, with knees slightly bent and a straight back posture (24). If needed, participants with poor balance may use a specifically designed adjustable metal frame for balance and support to ensure the patient can safely perform WBV (24). All sessions will be performed under the supervision of an experienced physiotherapist, part of the research team. A WBV diary will be maintained to record the patient’s progress, compliance with the study protocol, and any potential adverse events (including tiredness or pain) (24). Full adherence (100%) will be completion of all 36 sessions at the prescribed intensities and durations.

**Table 1:** The 12-week whole-body vibration training (WBV) protocol.

Exercise components		Week											
		1	2	3	4	5	6	7	8	9	10	11	12
Warm-up		5-minute stretching											
WBV	Frequency (Hz)	15	18	20	22	24	27	27	27	27	27	27	27
	Amplitude (mm)	2	2	2	3	3	3	3	4	4	4	4	4
	Vibration bout (min)	3	3	3	4	4	4	4	5	5	5	5	5
	Set (*n*)	3											
	Rest periods (min)	1											
Cool down		5-minute stretching											

The control group will attend supervised treadmill walking three times weekly for 12 weeks. The program will progressively increase in duration across the intervention period and will include stretching prior to and after each session. Walking speed and grade will be individually adjusted to reach the target intensity: participants with symptomatic PAD will walk until experiencing moderate claudication pain before resting and resuming, while those with asymptomatic PAD will walk continuously at a moderate intensity. In brief, both moderate intensities are assessed using subjective participant-reported measures. The moderate claudication pain in participants with symptomatic PAD is determined using a PAD-specific leg pain scale (26), a subjective pain intensity assessment tool. For individuals with asymptomatic PAD, moderate intensity is measured using the Borg Rating of Perceived Exertion (RPE) 6–20 scale (12, 13), a subjective exertion-based intensity measure, corresponding to moderate intensity as defined by the American College of Sports Medicine (ACSM) (27). The protocol was adapted according to Cochrane recommendations for standard walking therapy in PAD (11) (**Table 2**).

**Table 2:** The 12-week treadmill walking training protocol.

Exercise components			Week					
			1–2	3–4	5–6	7–8	9–10	11–12
Warm-up			5–minute stretching					
Walking	Frequency (sessions/week)		3					
	Time (min)		15	20	23	25	27	30
	Intensity	Symptomatic PAD	• Start at 2.5–3.0 km/h, 0% grade • Adjusting speed (0.2–0.5 km/h) or grade (1–2%) to elicit a moderate claudication pain (3–4/5 claudication scale) within 3–10 min					
		Asymptomatic PAD	• Start at 2.5–3.0 km/h, 0% grade • Adjusting speed (0.2–0.5 km/h) or grade (1–2%) to maintain a moderate intensity (RPE 11–13)					
Cool-down			5–minute stretching					

PAD, peripheral arterial disease; RPE, rating of perceived exertion (Borg scale).

### Primary outcome

The effectiveness of WBV will be assessed using the incremental shuttle walk test (ISWT) to examine changes in functional capacity (28). ISWT consists of 12 levels, with a total time signal of 12 minutes. Briefly, participants will be asked to walk on a 10-m long flat walkway following a beeping sound. The walking speed increased by 0.17 meters per second at one-minute intervals, beginning at 0.5 meters per second (level 1) and progressing to a maximum speed of 2.37 meters per second (level 12). The test will be terminated when participants cannot maintain the determined speed or meet the termination criteria following the exercise testing guidelines (29). The primary outcome will be the total distance covered during the test.

It should be noted that if a statistically significant beneficial intervention effect is observed, all patients in the control group will be offered the opportunity to undergo WBV.

### Secondary outcomes

#### Ankle-brachial index (ABI)

ABI will be evaluated using an automated ABI device (VaSera VS-1500 N, Fukuda Denshi, Tokyo, Japan), which has a moderate inter-rater reliability of measurement (intraclass correlation coefficient – ICC) of 0.7 (30). Participants will be required to remain in a supine position, resting for 10 minutes in a room with a constant temperature of 22 °C. Afterwards, systolic blood pressure will be measured twice on both brachial arteries and both posterior tibial arteries (with the systolic blood pressure not exceeding 10 mmHg) and ABI calculated as follows:


$$ABI\;\left(left\right)=\;\frac{\left(PTib1+PTib2\right)÷2}{hArm}$$

$$ABI\;\left(right\right)=\;\frac{\left(PTib1+PTib2\right)÷2}{hArm}$$

Where *PTib*1 and *PTib2* are the first and second systolic blood pressures of a posterior tibial artery, respectively, and *hArm* is the highest of the average value between right and left brachial artery.

#### Toe-brachial index (TBI)

TBI has moderate to good reliability (ICC = 0.75–0.77) (31) and will be measured as described above, except that lower limb measurements will be performed on each big toe, with TBI calculated as follows:


$$TBI\;\left(left\right)=\;\frac{\left(BT1+BT2\right)÷2}{hArm}$$

$$TBI\;\left(right\right)=\;\frac{\left(BT1+BT2\right)÷2}{hArm}$$

Where *BT1* and *BT2* are the first and second systolic blood pressures of a big toe, respectively, and *hArm* is the highest of the average value between right and left brachial artery.

#### Brachial artery flow-mediated dilation (FMD)

The FMD will be performed using a Doppler ultrasound device (Color Doppler ultrasound, Xario 100, Canon Medical Systems). With participants in a supine position, a blood pressure cuff will be positioned around the forearm and an ultrasound probe placed on the same arm proximally to the elbow, and their cardiac cycle will then be measured with a 3-lead electrocardiogram (ECG). A 30-second scan will be recorded as a baseline value prior to cuff inflation. Forearm ischemia will be applied by inflating to a pressure of 250 mmHg for 5 minutes. The cuff will then be deflated, and arterial images will be recorded 15 seconds after deflation and continue for three minutes. The maximum value of arterial diameter will be obtained by the average of 5 consecutive images of cardiac cycle (32). FMD will be calculated using the following equation (33):


$$FMD\;\left(\%\right)=\;\frac{Peak\;diameter-Baseline\;diameter}{Baseline\;diameter}$$

#### Glycated hemoglobin (HbA1C)

On the assessment day, a blood sample of 5 milliliters will be collected via venipuncture by a medical technologist.

#### Health-related quality of life (HRQoL)

HRQoL will be measured using the Thai version of the 36-Item Short Form Health Survey questionnaire (SF-36) (34), which consists of 36 questions encompassing eight subitems: physical function, role limitations due to physical problems, social function, bodily pain, general mental health (psychological distress and psychological well-being), role limitations due to emotional problems, vitality (energy/fatigue), and general health perceptions. The total SF-36 score ranges from 0 to 100, with a higher score indicating better HRQoL.

#### Disease-specific health status

Disease-specific health status related to PAD will be assessed using the Peripheral Artery Questionnaire (PAQ) (35). The PAQ consists of 20 items covering five subdomains: physical limitation, symptom stability, social limitation, treatment satisfaction, and quality of life. Scores range from 0 to 100, with higher scores indicating better disease-specific health status. A clinically meaningful change in the PAQ score is defined as 8 points or greater (36).

### Sample size calculation

The sample size was determined based on the primary outcome variable, maximum walking distance (MWD), as reported in a previous study by Zwierska et al. (37), which compared MWD between a leg exercise training group and a control (no exercise) group. Conservatively assuming a between-group difference of 50% of that reported by Zwierska et al., adjusting for baseline MWD, and using a two-sided α = 0.05 with 80% power, the required sample size was estimated at 25 participants per group. To account for a potential attrition of 15%, we aim to recruit 60 participants (30 per group). Sample size calculation was performed using the G*Power program (38).

### Adverse events

All potential adverse events will be recorded in the participant’s WBV diary (24). Mild to moderate adverse events that participants with PAD might experience as a result of WBV were identified from previous relevant studies (39–42), and include transient ones that resolve a few minutes after the WBV session (e.g., skin itchiness), although delayed onset muscle soreness (DOMS) due to the physical activity sessions will also be classified under this category (**Table 3**). Since WBV is a novel therapy for PAD, participants will be regularly monitored for the occurrence of any severe adverse events (**Table 3**), which could prematurely end the trial if likely associated with WBV. Adverse events will be assessed as objectively as possible and classified according to the Common Terminology Criteria for Adverse Events (CTCAE) v5.0 (39).

**Table 3:** List of potential adverse events.

Severity	Adverse event
Mild/Moderate	Delayed-onset muscle soreness (DOMS)
	Dizziness
	Drowsiness
	Falls
	Leg tingling
	Mild (temporary) headache
	Mild (temporary) knee pain
	Muscle fatigue
	Skin itchiness or redness
Serious	Arterial thromboembolism-related life-threatening consequences (hemodynamic or neurologic instability)
	Arterial thromboembolism-related death
	Hypertensive emergency (≥180/110 mmHg) requiring urgent intervention
	Hypertensive emergency-related death

### Blinding

Due to the nature of the intervention, it is not possible to blind participants to the treatment protocol they will be performing. In addition, it would be logistically challenging to attempt to blind the investigators overseeing the assessments, as it would rely on the participants not mentioning anything about their intervention.

### Data management

A REDCap database will be created for the study (43), as the software is secure and stored within Chiang Mai University servers. Study data will be entered directly into electronic case record forms (CRFs) in REDCap. Each form will be cross-checked by another member of our research team, with the final check performed by one of the senior investigators before the form is locked by the Data Management Unit at our Institute.

### Data reporting and statistical analyses

Demographic and clinical characteristics of participants at baseline will be reported for each group. The effectiveness of WBV on the primary outcome (ISWT) will be assessed on the intention-to-treat principle using all data collected on randomized participants. Data will be analyzed using a general linear model, adjusted for the participant’s age, sex, PAD categories (symptomatic or asymptomatic), diabetes duration, and the distance covered in the ISWT at baseline. The effect size will be reported as the adjusted between-group difference and its 95% confidence interval (CI). The potential interactions between randomization group and sex will be tested, and sex-specific differences reported as appropriate.

The same model structure will be used to examine the potential effectiveness of the intervention on secondary outcomes. Per-protocol analyses may be also carried out on primary and secondary outcomes excluding recipients with major protocol violations, in particular, lower levels of adherence (defined as completion of <67% of prescribed sessions).

Data will be analyzed using SAS v9.4 (SAS Institute, Cary, NC, USA) and/or SPSS v31 (IBM Corp, Armonk, NY, USA). Missing data on the primary outcome will not be imputed. All statistical tests will be two-sided at p<0.05, with no adjustment for multiple comparisons (44). Trial findings will be reported according to CONSORT 2010 guidelines (45).

## Ethics

Ethics approval for this study will be provided by the Research Ethics Committee at the Faculty of Associated Medical Sciences (Chiang Mai University). Written informed consent will be obtained from all participants. Further, this study will follow the appropriate institutional and international guidelines and regulations for medical research in accordance with the principles of the Declaration of Helsinki (46).

## Discussion

This study aims to address the current lack of evidence regarding the effectiveness of WBV training in individuals with T2D and PAD. Specifically, the study will examine the impact of WBV on functional capacity, vascular function, glycemic control, and quality of life. Findings are expected to provide important insight into whether (i) WBV training can improve functional capacity, and (ii) such improvements are attributable to augmented vascular function and better glycemic control in this population.

Previous literature has highlighted the close association between T2D and vascular health, with PAD being one of the most common and clinically significant complications of T2D (1). PAD in diabetic individuals is characterized by reduced blood supply to the lower limbs (2), and chronic vascular occlusion often contributes to intermittent claudication and consequent functional impairment. A number of studies have explored the effects of WBV training primarily on vascular function and glycemic control (14, 17–19) in various populations, including healthy adults and individuals with uncomplicated T2D. However, evidence addressing the clinical impact, particularly its effect on functional capacity (a meaningful and patient-centered endpoint in T2D individuals with PAD) remains limited. Thus, the present study has been designed to address this knowledge gap and to elucidate the potential clinical benefits of WBV training in this population.

Our WBV protocol was carefully designed based on parameters reported in previous studies, involving T2D individuals, which primarily investigated either vascular function (i.e., leg blood flow) or glycemic control (i.e., HbA1C) (19, 25) as outcomes. The current program was therefore developed to integrate and optimize the potential dose-response relationships of WBV, namely vibration frequency, amplitude, and duration to comprehensively target both vascular and metabolic adaptations. Lee et al. (19) demonstrated improved HbA1C following a 6-week WBV protocol using frequencies of 15–30 Hz, amplitudes of 1–3 mm, and exercise duration of 3x3 minutes. In contrast, Sanudo et al. (25) reported improved leg blood flow using a 12-week WBV program at a frequency of 12–16 Hz, 4 mm amplitude, and 12–20 minutes of exposure. Drawing on these findings, the current study protocol was designed to balance both intensity and duration to maximize vascular and glycemic benefits in T2D individuals with PAD. A randomized controlled trial design will minimize selection bias, ensuring the internal validity of the findings and reducing potential confounders. Furthermore, a comprehensive assessment approach, including measures of functional capacity, vascular function, glycemic control, and quality of life was implemented to capture multidimensional outcomes. This integrative evaluation is expected to bridge an important knowledge gap in understanding how WBV simultaneously influences these interrelated physiological domains.

Potential limitations in this study should be acknowledged. First, the single-center design may limit generalizability of the findings. Second, due to budget constraints, the study does not employ angiography, the gold-standard method for assessing vascular morphology to confirm PAD diagnosis. Instead, a validated functional assessment tool with acceptable diagnostic accuracy is utilized as an alternative (47). Despite these limitations, the methodological rigor of the study design, including randomization and comprehensive outcome evaluation, is expected to strengthen the internal validity and clinical relevance of the findings.

## Conclusion

This study is designed to investigate effect of WBV program training on functional capacity, vascular function, glycemic control, and quality of life in T2D individuals with PAD. Findings are expected to fill existing knowledge gaps regarding optimal exercise prescription for this population and to provide evidence-based guidance for clinical practice.
